# Fabrication of a Hard Tissue Replacement Using Natural Hydroxyapatite Derived from Bovine Bones by Thermal Decomposition Method

**Published:** 2014-02-01

**Authors:** E. Hosseinzadeh, M. Davarpanah, N. Hassanzadeh Nemati, S. A. Tavakoli

**Affiliations:** 1 *Department of Biomedical Engineering, Science and Research Branch, Islamic Azad University, Tehran, Iran.*; 2 *Iranian Tissue Bank and Research Center, Tehran University of Medical Sciences, Tehran, Iran*

**Keywords:** Compressive strength, Bovine bone, Defatting, Thermal decomposition, Bone allograft, Hydroxyapatite

## Abstract

Background: For the treatment of bone defects that exceed the critical size of the injury, several therapies have been investigated. Thermal decomposition method is suggested for extraction of natural hydroxyapatite bioceramic (HA). This technique in comparison with other methods of producing HA, has less complexity and greater economic efficiency.

Objective: In the present study, a thermal decomposition method is suggested for extraction of natural HA from bovine femur bones.

Methods: In this experiment, to extract the ceramic material, the bone samples were first de-fatted and ground to particles less than 420 μm, and also 420–500 μm, respectively. Prepared powders were heated at 170 °C for 24 h, and then divided into two groups for 6 h. The first group was heated at 750 °C; the second group was heated at 850 °C. The calcium phosphate compounds were obtained with complete elimination of the organic phase of the bone. These bioceramic compounds were characterized physiochemically by X-ray diffraction (XRD), fourier transform infrared spectroscopy (FTIR), energy dispersive X-ray (EDX), and scanning electron microscopy (SEM).

Results: We found that the powder heated at 750 °C in two dimensional scales was rich in carbonated hydroxyapatite, and therefore, eminently suitable for using in hard tissue replacements. However, increasing the temperature up to 850 °C reduced the Ca/P ratio to 1.5 in the powder sample sizes less than 420 μm. Consequently, the obtained composition became rather similar to the chemical formula of tricalcium phosphate (TCP) that is appropriate in tissue engineering and drug delivery applications.

Conclusion: The observations affirmed that by eliminating the collagen and other organic materials existing in the bovine bones, the mineral phase of the bone had the potential of transformation to nano-particles. To investigate the repair of critical-size bone defects and bone augmentation, cylindrical blocks were fabricated by applying different pressures of 150, 160 and 170 MPa. The structure and compressive strength of the pressed samples after sintering at 1200 °C were characterized by SEM and compressive strength test.

## INTRODUCTION

For the treatment of bone defects that exceed the critical size of the injury, several therapies have been investigated. Bone grafts are the most common approach. Autograft bone surgery is usually associated with lack of enough bone sources, infection, pain and suffering. In allograft surgery, the issue of disease transmission has always been a major concern. This means that an everlasting and growing demand exists for alternative biomaterials [1-3]. 

Today, bioceramics, due to their chemical and thermal stability, high strength and abrasion resistance, beautiful appearance and excellent biocompatibility, are suitable for use in hard tissues replacements. They are employed in various forms such as single crystal, polycrystalline, composites and coatings [4-10]. Calcium phosphates are compounds of great interest. They have been the subject of several investigations. Two principal representatives of this group, tri-calcium phosphate (TCP) and hydroxyapatite (HA), have large applications, particularly for biomedical purposes.

Hydroxyapatite, [Ca_10_(PO_4_)_6_OH_2_], the most thermodynamically stable calcium phosphate salt, is a compound of great interest in preparation of bioactive materials, mostly because it is the inorganic crystalline constituent in vertebrates calcified hard tissues such as bone and teeth. Given its participation in biological calcification processes, HA has been considered as the model compound for the physicochemical study of the biomineralization [4]. Unlike some of the calcium phosphate mineral phases such as alpha and beta tri-calcium phosphate which are more readily absorbed, the degradation rate is low. β-tricalcium phosphate (β-TCP) is represented by the chemical formula Ca_3_(PO_4_)_2_ with a Ca/P ratio of 1.5 [11].

Due to the escalating applications of HA in the body including facial reconstruction, orthopedics, ear, eye and dental implants [10], different processes for the production and manufacture of HA has been proposed [12]. Naturally, the priority is given to ways with more production ease at lower cost and higher efficiency. Extraction of HA from the bones, where it is abundant, is the simplest and most cost-effective technique and has a higher biological safety, compared to other methods.

Three methods have so far been reported for this type of extraction: 1) Subcritical water process where the organic phase of the bone powder is eliminated by immersion of the bone in deionized water at 250 °C; the mineral phase is recovered after drying [13]; 2) alkaline hydrothermal hydrolysis where the hydrothermal experiment has been achieved by applying sodium hydroxide solution at a concentration of 25% (wt) at a temperature of 250 °C for 5 h that destroys the organic phase [13]; and, 3) thermal decomposition where the organic phase of the bone is lost through heat treatment; the mineral phase can be characterized. 

In this study, we addressed the thermal decomposition method. The high temperature treatment of this technique can remove every genome sign of each disease. Therefore, it has a high biological safety factor. Less complexity and greater economic efficiency are other advantages of this method.

To investigate the repair of critical-size bone defects and bone augmentation, cylindrical blocks were sintered by applying different pressures of 150, 160 and 170 MPa. The structure and compressive strength of the pressed samples after sintering at 1200 °C were characterized by scanning electron microscopy (SEM) and compressive strength tests.

## MATERIALS AND METHODS

Bovine bone preparation 

**Figure 1:**
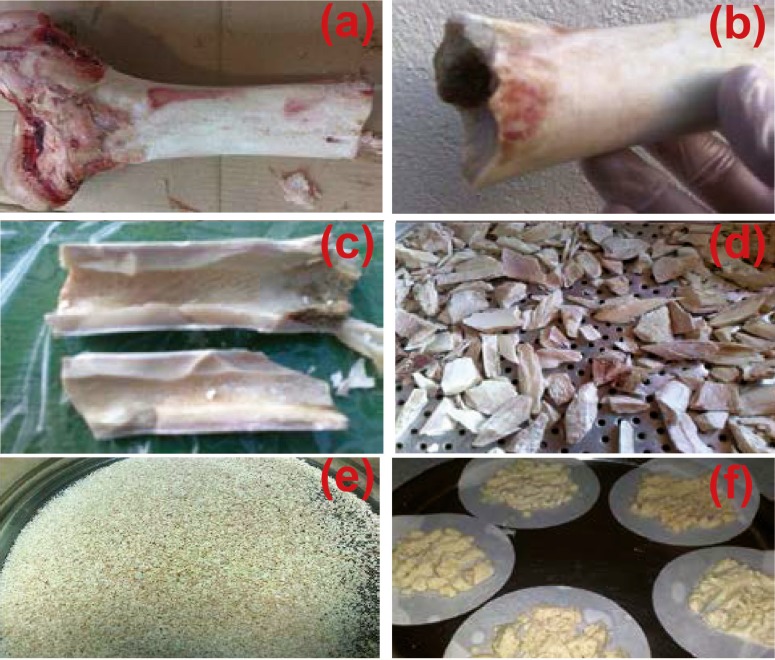
Bone powder preparation steps in order to extract hydroxyapatite: a) cutting, b) separation of soft tissue, c) removing of bone marrow, grinding d) first e) second (powder production) and f) prepared powders

First, the bovine femur cortical bone was selected and the soft tissue and the periosteum were removed. The medullary portion of the bone, bone marrow and other soft tissues were also evacuated. In the second step, for removing fat, the bone was chopped into smaller pieces and dissolved in chloroform (Merck, Germany). Then, the pieces were dried and crushed again by a crushing medical device (IKA, Grinder, Germany) with a 500-µm filter. The produced powders were passed through a 420-µm mesh. Finally, after removing the fat, bone powders were sunk in acetone (Merck, Germany) for 2 h and then washed with distilled and deionized water three times. Powders were removed by filter paper from the washing liquid. The preparation steps are depicted in Figure 1. The manufactured powders were dried in the oven (Memmert, Germany) at 170 °C for 24 h. The bone meal was categorized in two groups according to the size of particles—the first group included particles smaller than 420 µm and the second group consisted of particles sized between 420 and 500 µm. The particles were prepared for heat treatment. The particles which passing 420 µm meshes size are in the same dimensional range than the second group. It can be considered in sintering process. 

Thermal decomposition 

The bone powder became yellowish brown after drying in the oven. The powder did not receive any further heat treatment; these samples were labeled as raw bone powder. In the thermal decomposition process, a 10-g sample was placed in an open alumina crucible and then heated in the furnace (Atbin, Iran) at 750 °C for 6 h; the same amount of powder was heated in the same condition at 850 °C. The bone powder became white after the heating stage.

Sintered blocks preparation 

To build cylindrical specimens (φ 5×10 mm^2^), the HA powder prepared in the previous step was utilized. To examine the feasibility of these blocks for replacing in the hard tissue, they were compared with some commercial bone allografts produced in Iranian Tissue Bank-Research and Preparation Center, Imam Khomeini Hospital, Tehran, Iran. HA powders were pressed in a stainless steel mold using a hydraulic press (Gotech, Taiwan) in three different pressures 150, 160, 170 MPa. In order to achieve porosity and proper lubrication, HA powders were mixed with liquid paraffin (2 mL) and naphthalene (%v) before being pressed. Finally, the blocks were sintered at 1200 °C for 2 h in the furnace (Exciton, Iran). The rate of increasing the furnace temperature was set at 10 °C/min.

Characterizations 

Fourier transform infrared spectroscopy (FTIR) (Termo-Nicolet FT-IR) was used to study the absorption of infrared beams across the prepared powders. Surface morphology was studied by a (VEGA/TESCAN) SEM equipped with energy dispersive X-ray (EDX). The phase, crystallinity and particle size were characterized using an X-ray diffractometer (Seifert 3003PTS) with radiation over a range of 2θ angles from 20° to 80°. After heating, the specimens were ground by a mortar. Compressive strength of the samples was also measured using the pressure test (SANTAM-STM-50, Iran).

## RESULTS AND DISCUSSION

XRD results 

**Figure 2:**
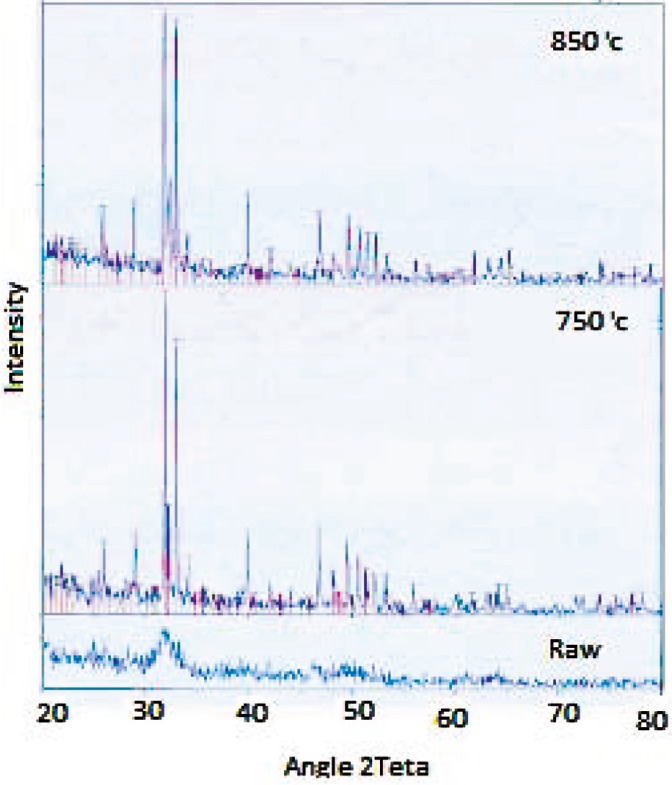
XRD spectra of raw bone powder sample and heated samples at 750 and 850 °C for 6 h.

XRD analysis is a highly trustable means used to probe the crystalline compounds. Figure 2 shows the XRD spectra of the raw bovine bone and the bone powder samples treated at 750 and 850 °C. All peaks corresponding to the standard spectrum HA were observed in both heated samples. However, this picks were not observed in the XRD spectra of the raw powder samples and the amorphous structure was conspicuous. This is because the collagen and other organic materials were retained despite applying the heat (24 h at 170 °C). It could be said that the initial heating evaporated the water just to dry the bone powder. Table 1 shows the planar spacing (estimated by the Bragg's law) and the intensities at the strongest peaks in the XRD spectra and their comparison with the standard HAp data (JCPDS) [14]; the error was estimated at each plane.

**Table1:** planar spacing and intensities obtained from X-ray diffraction of heated samples, the results are compared with the standard HAp (JCPDS)^[14]^.

	**d (nm)**			**Intensity**		
**Hkl**	**JCPDS**	^o^C750	^o^C850	**JCPDS**	^o^C750	^o^ **C** **850**
002	440/3	433/3	449/3	40	23	29
211	814/2	809/2	819/2	100	100	100
112	720/2	715/2	720/2	60	85	96
202	631/2	626/2	635/2	25	19	19
310	262/2	260/2	264/2	20	26	34
222	943/1	942/1	946/1	30	28	27
321	806/1	805/1	807/1	20	18	21
004	722/1	720/1	732/1	20	13	5
311	148/2	146/2	152/2	10	10	14
501	587/1	586/1	732/1	4	5	5
102	170/3	170/3	174/3	12	11	12
Error		1/110 × 10^-3^	9/892× 10^-3^		202/0	305/0

By comparing the heated samples we can see that the HA peaks corresponding to the angles of almost 26°, 30° and 35° [15], are higher and less widely in heated samples at 750 °C. So the HA phase is more significant in these samples. In contrast, the peak corresponding to the β-TCP phase θ_2_ equal angles about 27.7°, 44.5° and 47.7° [15] are found to be higher and less widely in heated samples at 850 °C. It can be concluded that high temperature led to disintegration of HA phase to the β-TCP phase.

Ooi, *et al* [16], stated that HA to TCP transition is not happened up to temperature of 1000 °C. Nevertheless, this study shows that with increasing time of heating, lower temperatures (*eg*, 850 °C) can also lead to catalysis of HA to TCP.

FTIR results 

**Figure 3:**
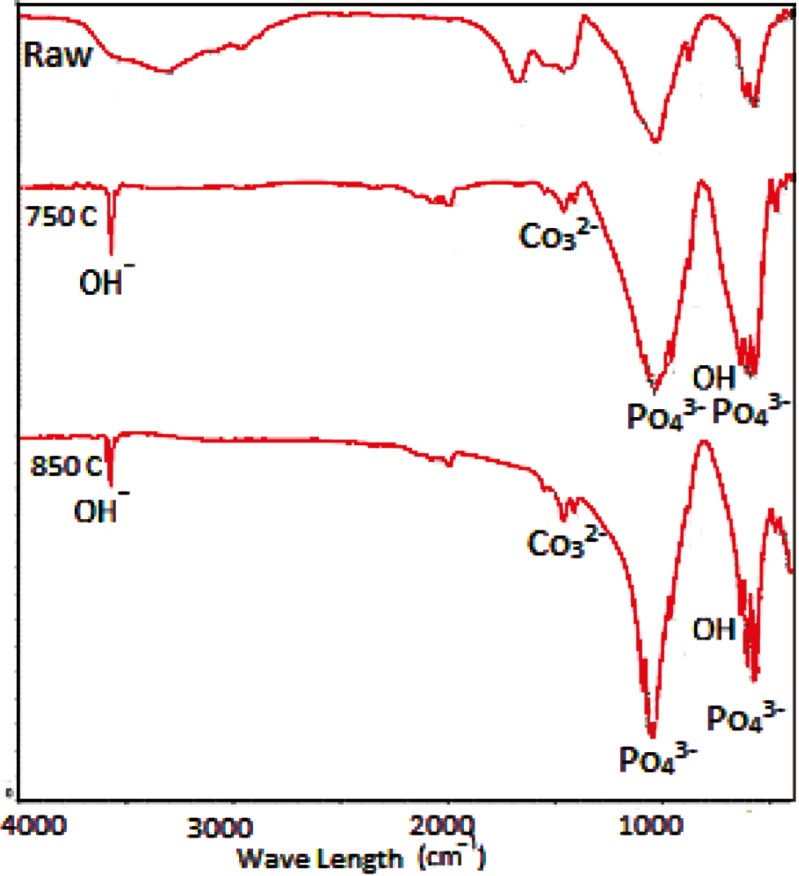
FT-IR for raw bone powder sample and heated samples at 750 and 850 °C for 6 h.

FTIR is an excellent tool for structural investigations because of the knowledge of the vibration origins of the amide bonds, the sensitivity of some of these band positions to conformation, and the possibility of predicting band positions for a given helical or extended conformation [13]. Figure 3 demonstrates the FTIR spectra from three samples of raw, and heated at temperatures of 750 and 850 °C. Differences between the spectra of raw bones and two other samples (750 and 850 °C) are due to changes in their chemical structure, occurred during the heating. The spectra of heated samples are more similar to the standard spectra of pure HA [9], implying that the temperature destroys collagen and other structural proteins of the bone [16]. Two picks at 2964 and 1678 cm^−^^1^ corresponding to NH and amid bands that are observed in the raw bone spectra, are completely eliminated in heated samples. This fact indicates the presence of the proteins and the organic phase in raw bone samples and their absence in two heated samples. Burning and destroying all bone proteins and the organic phase is confirmed by the change in the color of the powders from yellowish brown (raw) to white (heated) [16]. 

Due to the high temperature phase of HA, by increasing the temperature, we expect to have the OH band as a sign of the HA phase. As shown in Figure 3, the intensity of the OH stretching band in two heated samples at 750 and 850 °C are 3572 and 3571 cm^–1^, respectively. The OH bending band is also shown at 630 cm^–1^. However, there is no OH band in raw bone powder spectra. The intensity of this peak is decreased with increasing temperature up to about 1000 °C [15]. At this temperature, HA is dehydroxylated, its thermal stability is lost and β-TCP is produced [9, 16]. The following reaction is carried:

Ca_10_(PO_4_)_6_(OH)_2_   3β-Ca_3_(PO_4_)_2_ + CaO +H_2_O (1)

This catalysis reaction takes place at 850 °C. This hypothesis can be attributed to the increase in the heating time.

According to the formula of Ca_10_(Po_4_)_6_(OH)_2_, phosphate is an essential component of the structure of this material. Some sharp peaks at the wave numbers of 500 cm^−^^1^ up to 1100 cm^–1 ^in the two heated samples spectra in ure 3 are assigned to the P-O band. The peak intensity increases with increasing the temperature, which represents a large amount of phosphate bands in the powder at 850 °C. By increasing phosphates, the Ca/P ratio decreases and its results are noticeable in EDX analysis.

Carbonate groups at wave numbers 2000, 1400 and 900 cm^–1 ^are seen in Figure 3. By increasing the heating temperature from 750 to 850 °C, the intensity of this group increases slightly. The longer duration of heating at 850 °C (6 h) leads to the appearance of the carbonate groups. Carbonated HA has a significant role in biomedical application. Therefore, by decreasing the duration of heat treatment, carbonate groups may be lost [17].

Scanning electron microscopy (SEM) 

**Figure 4:**
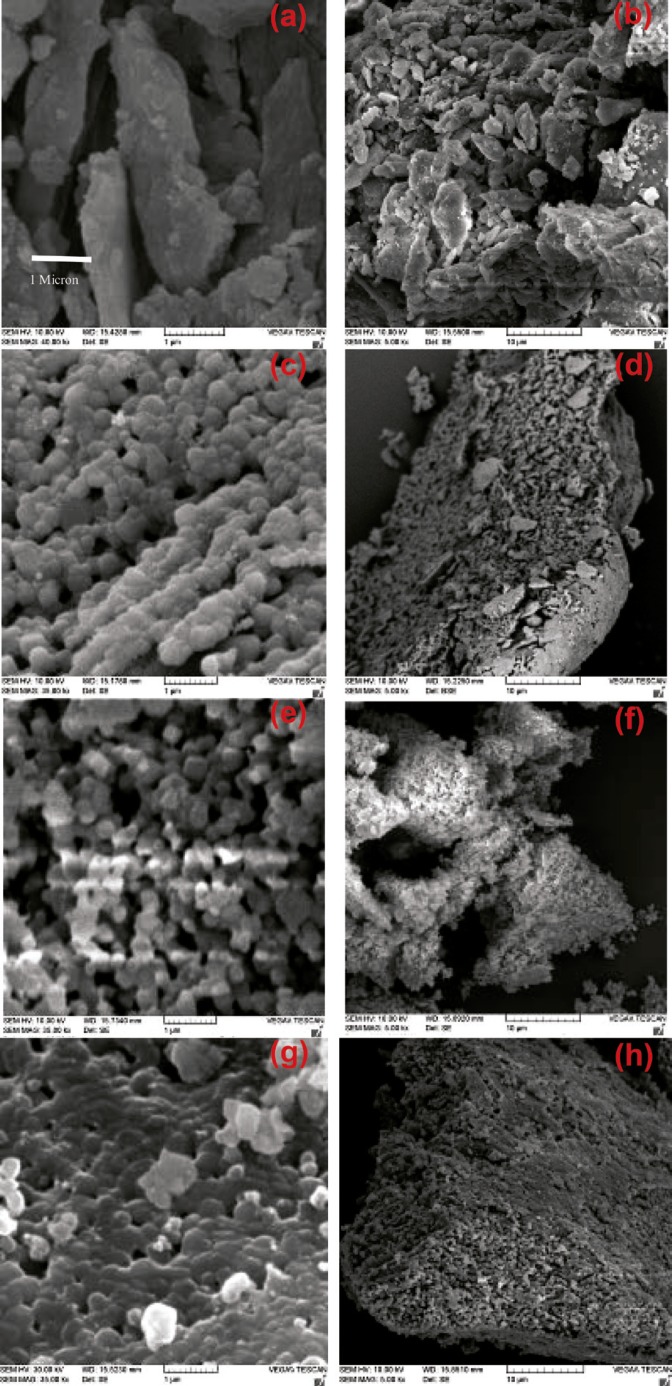
SEM images for a) and b) bone crushing blades; c) and d) heated bone crushing blades at 750 ºC; e) and f) milled powder samples after heat treatment at 750 ºC; and g) and h) at 850 ºC.

SEM was used to evaluate the morphology of the obtained HA. SEM images of the prepared powders are shown in Figure 4. Figures 4a and 4b show crushed bone which was confirmed to have both organic and mineral phases on FTIR. Heat treatment eliminates the organic phase of these blades and makes them porous (Figures 4c and 4d; 750 °C). The structural integrity of the bone can be destroyed by the removal of the collagen which makes it very fragile in a way that is ground into powder by the lowest force (Figures 4e and 4f). Accurate observations of the morphology of heated powders showed that they were in a nanometer scale. This issue was observed in the powder samples annealed at 850 °C, too (Figures 4g and 4h). The difference between these two recent powder examples was in the Ca/P ratio that was reflected in EDX results.

**Figure 5:**
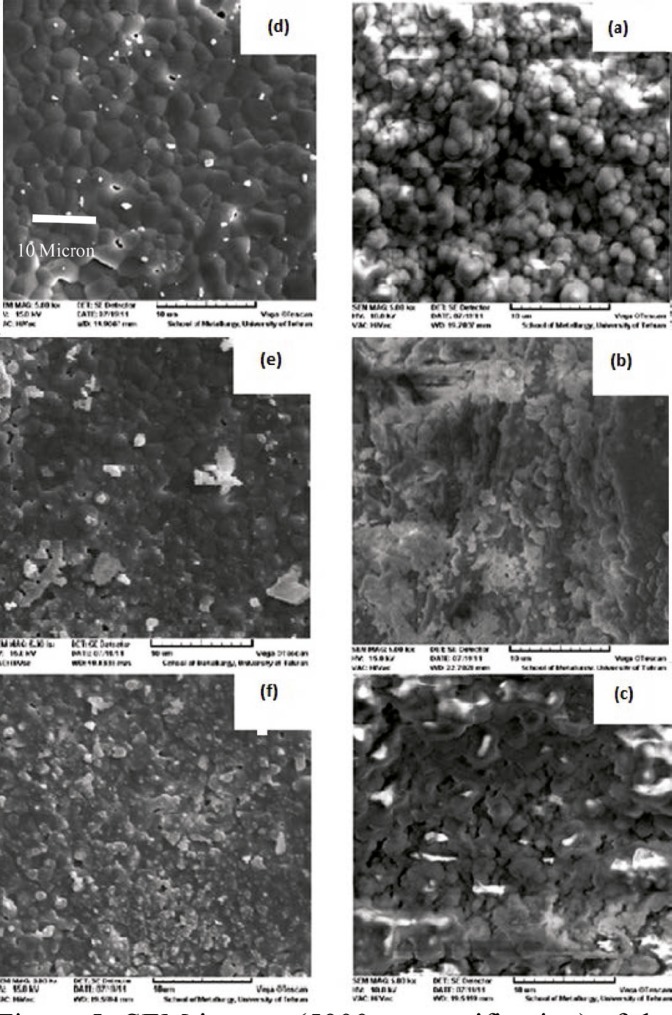
SEM images (´5000 magnification) of the raw blocks pressed at pressures a) 150, b) 160, and c) 170 MPa and sintered blocks at pressures d) 150, e) 160, and f) 170 MPa

**Figure 6:**
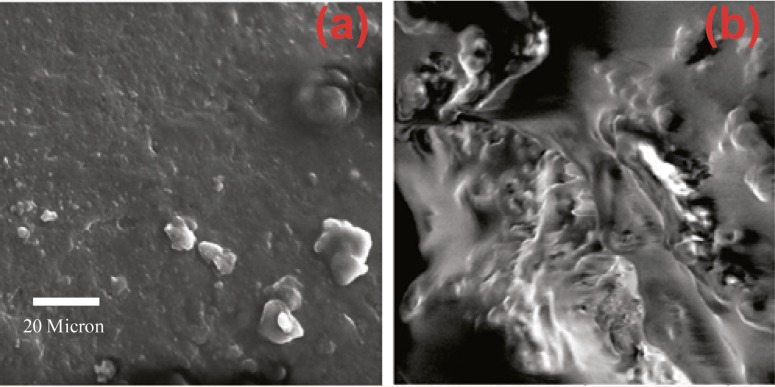
SEM image of a bone allograft a) cancellous bone, b) cortical bone. (×3000 magnification)

SEM images of produced HA blocks made ​before and after sintering are shown in Figure 5. By increasing the impact force, powder particles became more compact and dense. Liquid paraffin and naphthalene in samples were removed through the sintering process. Surface melting happened to HA particles, causing the particles to weld to each other and to represent necking in particle junctions. The process of sintering resulted in finer grains, and uniformity and greater density of the samples. Therefore, the structure of sintered HA block in high pressure was more close to cortical allografts. For better comparison, surface morphology of bone allograft samples were examined by SEM (Fig 6).

EDX results 

**Figure 7:**
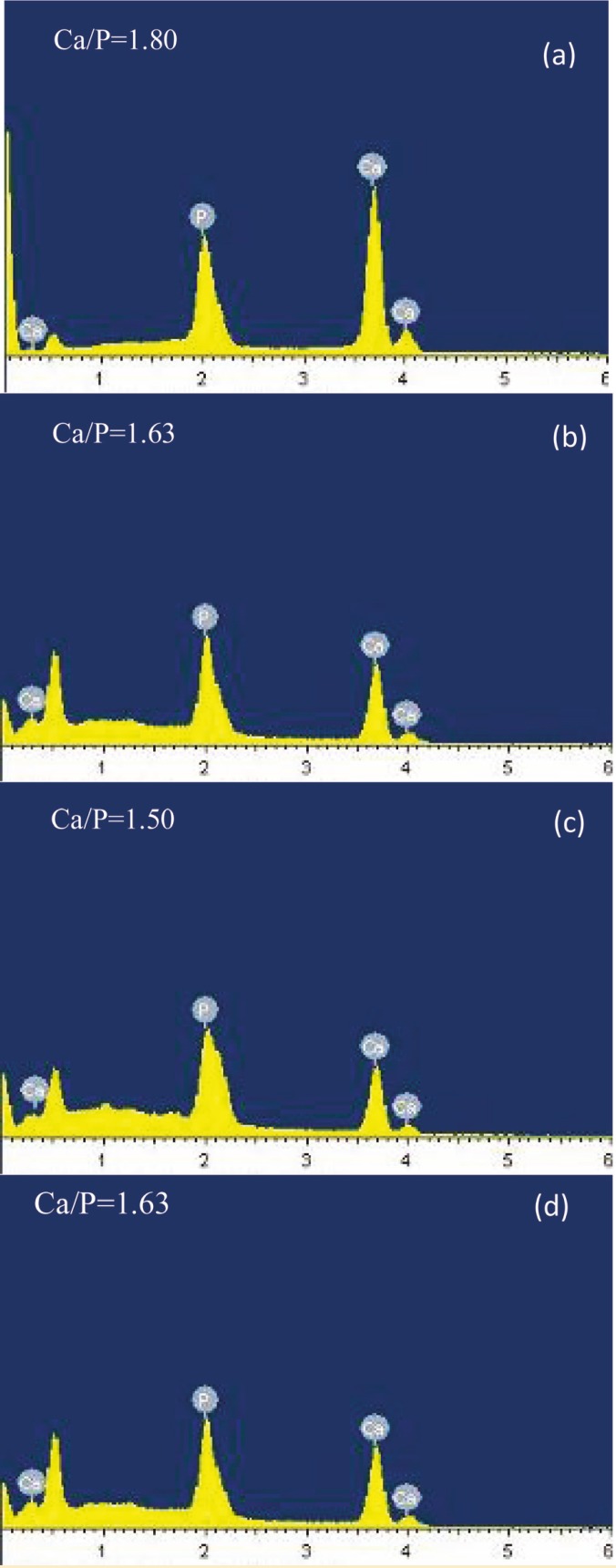
EDX results for powder samples smaller than 420 µm, a) raw, heated at b) 750 ºC d) 850 ºC and e) powder samples in the range of 420–500 µm and heated at 850 °C.

The results of EDX analysis are shown in Figure 7. According to the chemical formula of HA, calcium and phosphorus, the most significant minerals of the bone, are noticeable in this analysis. The calcium to phosphorous molar ratio was 1.67 in HA and 1.5 in β-tricalcium phosphate. From this ratio (Figure 5), for each sample, it can be concluded that heating leads to a lower ratio when compared to the raw samples. The results of fine samples (<420 µm) heated at 750 ºC and coarse samples (420–500 µm) heated at 850 ºC, are closer to the stoichiometric HA standard ratio. The minimum Ca/P ratio is related to small samples heated at 850 °C, indicating that the powder combination has β-TCP phase.

**Figure 8:**
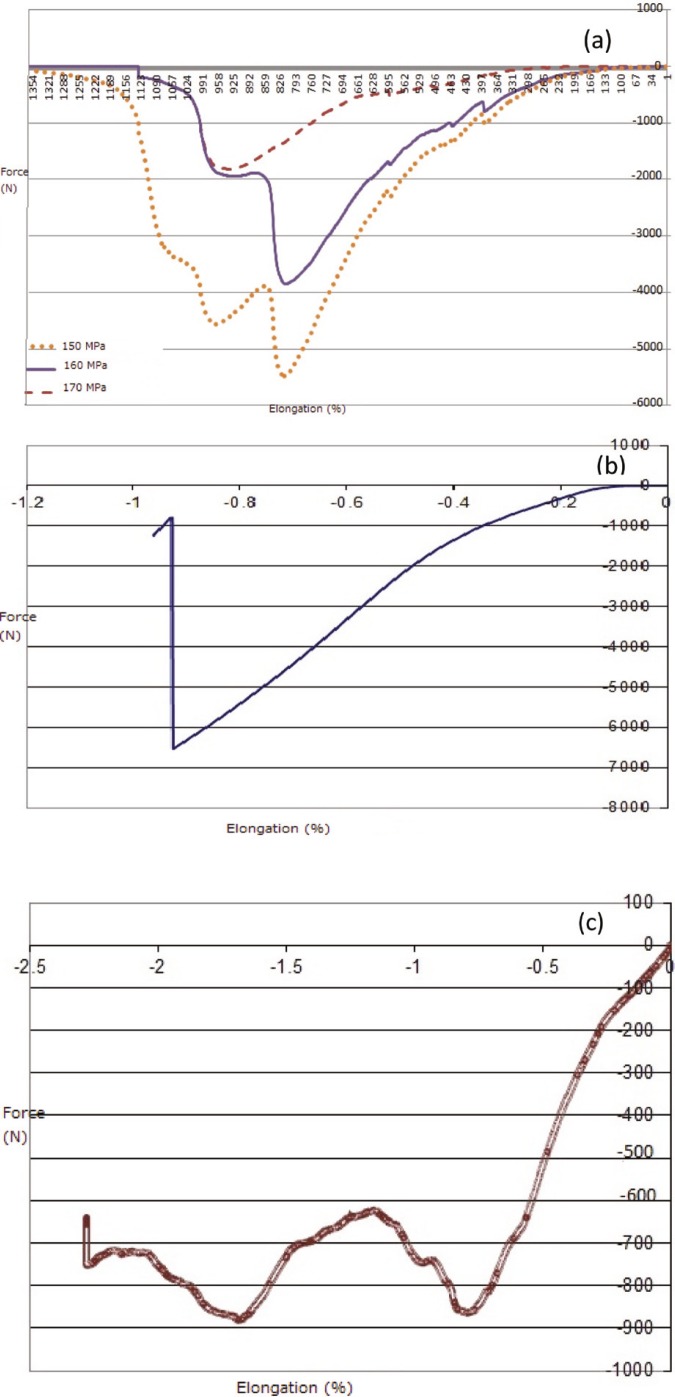
Compressive strength results as force-elongation curve for a) sintered and pressed samples at 150 Mpa, 160 Mpa, 170 Mpa, and commercial bone allografts; b) cortical bone, and c) cancellous bone.

Compressive strength test results 

To obtain and compare the resistance of sintered HA and bone allograft blocks towards the compressive force applied to them, the compressive strength test was used. Compressive strength test results for pressed samples are shown schematically in Figure 8. 

All the samples followed a similar pattern. All the curves had both elastic and plastic regions—a plateau region related to collapsing of the internal porosities and at the end broke down where they endured the utmost stress. 

Regarding the cancellous allograft curve (Fig 9), it was observed that the samples had no break down stage and there were only elastic and plastic stages. As the cancellous samples had no break down and only collapsed, the pressure was applied to the samples until they crushed and force application was then stopped. Since break down was replaced by crush, we were not able to determine the compressive strength and break down strain for cancellous samples but according to the literature, we regarded 2–12 MPa as the compressive strength of cancellous bones.

According to the results mentioned in Table 2, the produced blocks can be used as bone replacements, especially cancellous allografts.

**Table 2:** The results of compressive strength tests

**Elastic Modulus (Mpa)**	**Compressive Strength (Mpa)**	**Samples**	
41.87	5.94	Cancellous allograft	
89.80	56.20	Cortical Allograft	
		Mpa	Sample
42.43	10.82	150	1
56.72	17.56	160	2
76.59	23.41	170	3

## CONCLUSION

Thermal decomposition is an easy and affordable way to extract HA from useless resources; due to its biocompatibility properties and normal ossification conduction, it will have a high potential to replace in the human body. Observations showed that heating at 750 ºC for 6 h extracts HA from the bovine femur. In the case of coarse particles (420–500 µm), this finding is observed after heating at 850 ºC. This product has important medical applications. Heat treatment of bone samples with grains <420 µm at 850 ºC made calcium phosphate compounds rather similar to TCP for use in biodegradable applications. This can be inferred that all these three parameters, temperature, heat treatment time, and size of bone powder particles, are involved in determining the composition of the extracted calcium phosphate and consequently its application.

By comparing the greatest strength of HA blocks with cortical and cancellous allograft samples, it could be concluded that the samples pressed in the mold with more pressure had more strength and are more similar to cortical allografts. According to statistical analyses, we believe that the samples pressed at 170 Mpa can appropriately be utilized in small bone defects with low loading condition as an allograft replacement. 
